# A Novel Prior- and Motion-Based Compressed Sensing Method for Small-Animal Respiratory Gated CT

**DOI:** 10.1371/journal.pone.0149841

**Published:** 2016-03-09

**Authors:** Juan F. P. J. Abascal, Monica Abella, Eugenio Marinetto, Javier Pascau, Manuel Desco

**Affiliations:** 1 Departamento de Bioingeniería e Ingeniería Aeroespacial, Universidad Carlos III de Madrid, Madrid, Spain; 2 Instituto de Investigación Sanitaria Gregorio Marañón (IiSGM), Madrid, Spain; 3 Centro de Investigación en Red de Salud Mental (CIBERSAM), Madrid, Spain; North Shore Long Island Jewish Health System, UNITED STATES

## Abstract

Low-dose protocols for respiratory gating in cardiothoracic small-animal imaging lead to streak artifacts in the images reconstructed with a Feldkamp-Davis-Kress (FDK) method. We propose a novel prior- and motion-based reconstruction (PRIMOR) method, which improves prior-based reconstruction (PBR) by adding a penalty function that includes a model of motion. The prior image is generated as the average of all the respiratory gates, reconstructed with FDK. Motion between respiratory gates is estimated using a nonrigid registration method based on hierarchical B-splines. We compare PRIMOR with an equivalent PBR method without motion estimation using as reference the reconstruction of high dose data. From these data acquired with a micro-CT scanner, different scenarios were simulated by changing photon flux and number of projections. Methods were evaluated in terms of contrast-to-noise-ratio (CNR), mean square error (MSE), streak artefact indicator (SAI), solution error norm (SEN), and correction of respiratory motion. Also, to evaluate the effect of each method on lung studies quantification, we have computed the Jaccard similarity index of the mask obtained from segmenting each image as compared to those obtained from the high dose reconstruction. Both iterative methods greatly improved FDK reconstruction in all cases. PBR was prone to streak artifacts and presented blurring effects in bone and lung tissues when using both a low number of projections and low dose. Adopting PBR as a reference, PRIMOR increased CNR up to 33% and decreased MSE, SAI and SEN up to 20%, 4% and 13%, respectively. PRIMOR also presented better compensation for respiratory motion and higher Jaccard similarity index. In conclusion, the new method proposed for low-dose respiratory gating in small-animal scanners shows an improvement in image quality and allows a reduction of dose or a reduction of the number of projections between two and three times with respect to previous PBR approaches.

## Introduction

**Fig 1:**
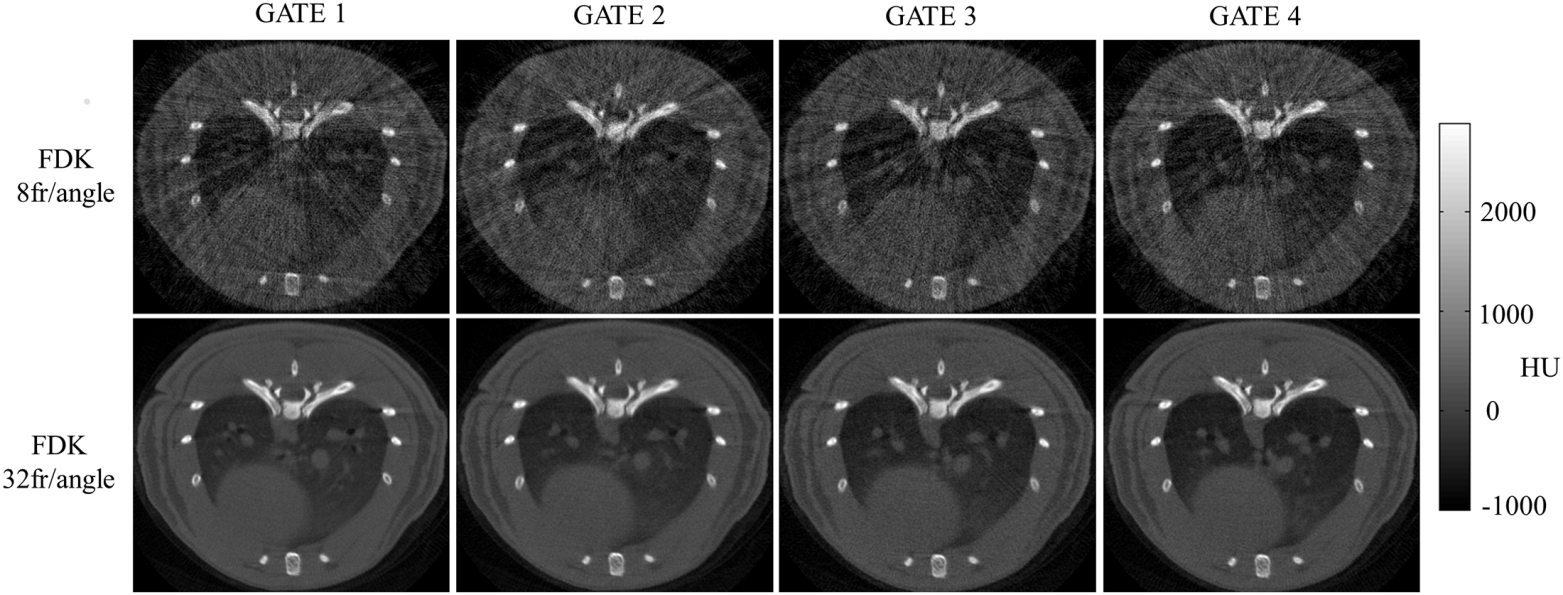
Standard dose vs. versus high dose respiratory gated data. Top: FDK reconstructions of gated data with four time points over the breathing cycle (four gates) obtained with a standard protocol for static studies (360 views covering 360° with eight frames per projection angle). After assigning frames to different gates, few and irregularly distributed projections are left for the reconstruction of each respiratory phase. Bottom: FDK reconstructions of gated data obtained with ideal high-dose data comprising 32 frames per projection angle, which is four times the dose of the standard protocol.

Respiratory gating helps to overcome the problem of breathing motion in cardiothoracic small-animal imaging. This may be relevant in several clinical situations. For instance: 1) when using the CT for attenuation correction in PET [1]; 2) when assessing the degree of infection in lung diseases as tuberculosis, which is generally based on the inspection of images to quantify the density and extension of nodules in the lung [2]; 3) when planning radiation therapy for treatment of tumors in chest and abdomen, in which movement due to normal breathing complicates tumor location [3,4]. We use the term gate to denote each different reconstructed image for different time points over the breathing cycle. One option to generate a gated study is to acquire multiple frames from every projection angle, each one corresponding to a different point over the breathing cycle, and to sort them out assigning each frame to the corresponding gate according to a respiratory signal [5]. To achieve good image quality for each respiratory phase with this approach, conventional reconstruction methods require more data than those actually acquired with a standard protocol for static images. As an example, top panel of Fig 1 shows the effect of sorting data into four gates when using the dose of a standard static protocol: after assigning frames to different gates, few noisy and irregularly distributed projections (less than 8 frames/projection) are left for the reconstruction of each respiratory phase, leading to streak artifacts in the FDK reconstructed images. Bottom row in Fig 1 shows the result for a high-dose protocol (four times more frames per projection angle, if reconstructing four respiratory gates).

Since radiation can affect the immune system and modify other biological pathways, dose must be kept low, particularly in longitudinal studies [6]. Besides, the ultimate goal of these techniques is to be translated to the clinical field, where the ALARA principle ('As Low As Reasonably Achievable') holds for the patient radiation dose (http://www.nrc.gov/reading-rm/basic-ref/glossary/alara.html).

Compressed sensing enables accurate image reconstruction from few projections using convex optimization, provided that the image is sparse in a transformed domain [7,8,9,10,11]. Prior-based reconstruction (PBR) is widely used for CT since reconstructed images are highly sparse when subtracted from a prior image that, in the case of dynamic applications, can be obtained by averaging all the data.

The most widely used PBR method is prior image constrained compressed sensing (PICCS), which has been widely tested in different applications [12,13,14,15,16]. The image gradient is the most commonly used transformed domain but, as we showed in a previous study, using a wavelet transform in the prior term instead of the gradient leads to better texture for PICCS [16]. This type of prior is free from artifacts but is blurred due to temporal averaging.

Motion-based reconstruction obtains an even sparser transformed domain by reconstructing images using an estimation of motion between consecutive frames. This approach has been proposed for cardiac cine MRI [17,18,19].

Few previous studies have combined prior-based reconstruction methods with registration in CT [20,21,22,23]. These works aimed to improve image quality in low dose acquisition using a previously acquired high-quality prior image not spatially registered with the data, thus requiring a registration step which was implemented by modifying the prior penalty term. However, these methods would not work for respiratory gating in small-animal CT because of two main reasons: first, there is no a high quality previous acquisition image; second, the prior is a blurred image made from averaging all gates.

In this work, we propose a novel prior- and motion-based reconstruction (PRIMOR) method for respiratory gating in small-animal CT, which extends the PBR method in [16] by including a model of the motion between gates. A prior image is obtained as the average of all respiratory gates reconstructed with an FDK-based algorithm. Motion is estimated using a nonrigid registration method based on hierarchical B-splines. We compared PRIMOR and PBR on different simulated scenarios, created by changing the number of photons (dose) and the number of projections from a reference FDK reconstruction of high dose data previously acquired with a micro-CT scanner. In both cases the problem was solved using the Split Bregman approach, which is efficient for convex constrained optimization [24].

## Methods

### Image Reconstruction

#### PBR

Gated CT images can be accurately reconstructed from highly undersampled and noisy data using PBR methods, such as PICCS. These methods assume that each respiratory phase image *u*_*i*_ is sparse under the transformation Ψ, which accounts for spatial sparsity, and that there must be a prior image ${\bar{u}}_{p}^{}$ to ensure that the difference image obtained by subtraction of each gate from the prior, $u_{i}-{\bar{u}}_{p}^{}$, is sparse under the transformation Φ [12]. If *f*_*i*_ represents the data corresponding to the *i-*th gate image and *F* is the forward operator, PICCS solves the convex constrained optimization problem

$$\underset{u}{\text{min}}\beta \;{\left\|\Psi \left(u\right)\right\|}_{1}+\;\alpha {\left\|\Phi (u-u_{p})\right\|}_{1}\;\text{such}\;\text{that}\;{\left\|Fu-f\right\|}_{2}^{2}\leq \sigma^{2}$$ (1)

where *u* represents the reconstructed gates and *f* represents the acquired data for all respiratory gates, i.e. *u* = [*u*_1_^T^,…,*u*_I_^T^]^T^, *f* = [*f*_1_^T^,…,*f*_I_^T^]^T^, $u_{p}={\left[{\bar{u}}_{p}^{T},\ldots ,{\bar{u}}_{p}^{T}\right]}^{T}$, *I* is the total number of respiratory phase bins, σ accounts for noise in the data, β weights the image penalty function and α weights the prior penalty function.

In this work, we use an equivalent of Eq (1) to recover only the image variation with respect to the prior for each gate, *v* = [*v*_1_^T^,…,*v*_I_^T^]^T^. Thus, we define the PBR method used in this work,

$$\underset{v}{\text{min}}\beta \;{\left\|\Psi \left(u_{p}+v\right)\right\|}_{1}+\;\alpha {\left\|\Phi (v)\right\|}_{1}\;\text{such}\;\text{that}\;{\left\|F(u_{p}+v)-f\right\|}_{2}^{2}\leq \sigma^{2}$$ (2)

The common choice for Ψ is the spatial discrete gradient that leads to TV, ‖∇*u*‖_1_, which filters out noise while preserving edges in the image. We adopt isotropic TV, ${\left\|\nabla u\right\|}_{1}=\sqrt{{\left(\nabla_{x}u\right)}^{2}+{\left(\nabla_{y}u\right)}^{2}}$. For Φ we use the symlet wavelet transform [25] to impose sparsity on the image variation, which was previously found to provide a more natural texture than TV for the prior term [16].

#### PRIMOR method

**Fig 2:**
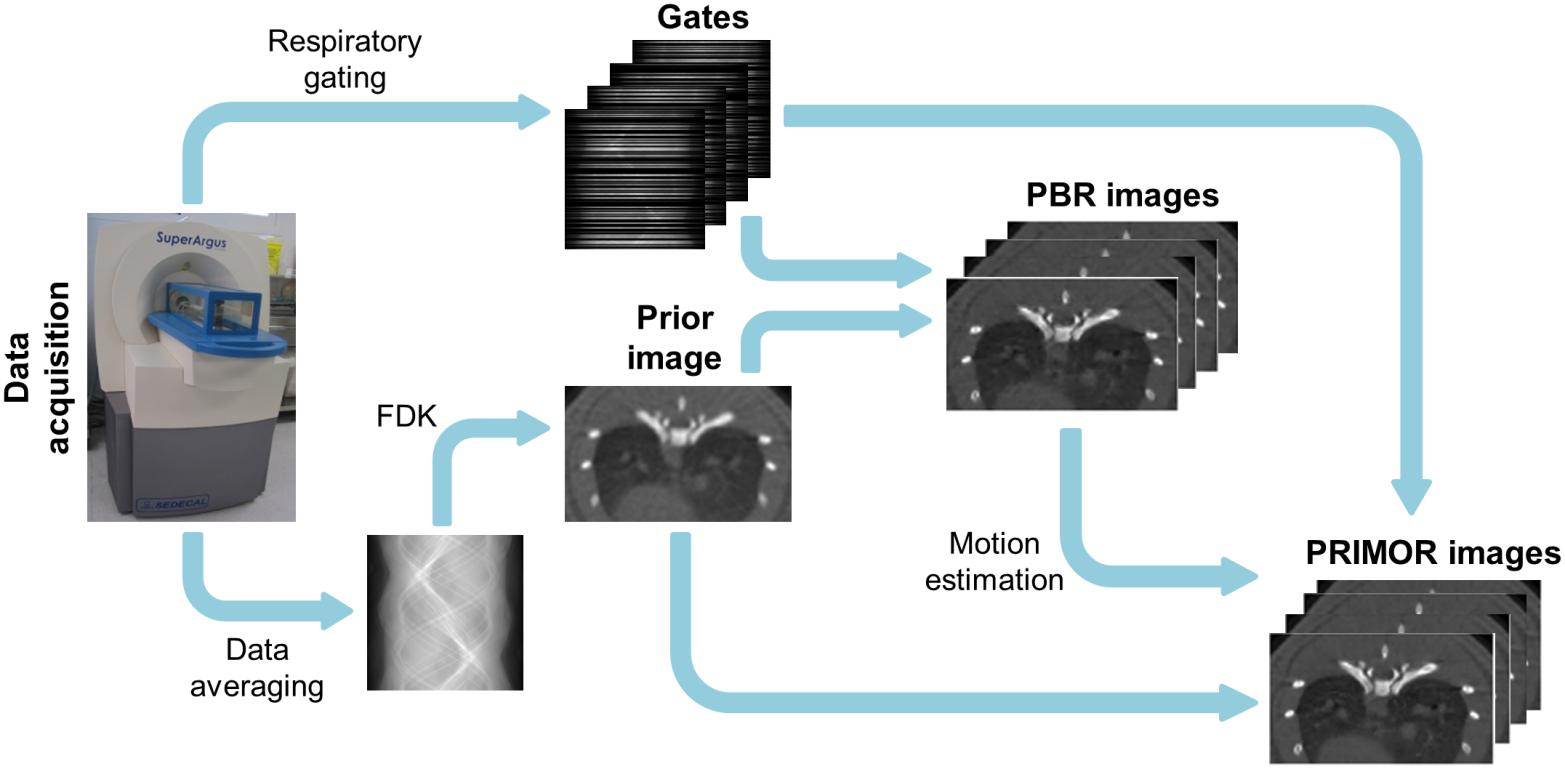
Workflow of PRIMOR reconstruction.

PRIMOR improves PBR by adding a penalty function that takes into account a previously computed model of motion. If *T* represents an operator that encodes the motion between consecutive gates, we define PRIMOR by including the term ‖*T*(*u*_*p*_ + *v*)‖_1_ in PBR [Eq (2)] with the weighting parameter γ

$$\underset{v}{\text{min}}\beta {\left\|\Psi (u_{p}+v)\right\|}_{1}+\;\alpha {\left\|\Phi v\right\|}_{1}+\;\gamma {\left\|T(u_{p}+v)\right\|}_{1}\;\text{such}\;\text{that}\;{\left\|F(u_{p}+v)-f\right\|}_{2}^{2}\leq \sigma^{2},$$ (3)

where the prior image ${\bar{u}}_{p}^{}$ is defined as the average of all respiratory gates reconstructed with an FDK-based algorithm [26]. A sketch of the method is shown in Fig 2.

For the motion estimation, the temporal sparsity transformation *T* can be computed in terms of nonrigid registration between frames as

$$Tu=\left[\begin{array}{c} u_{1}-R_{1}u_{I}\\ u_{2}-R_{2}u_{1}\\ \ldots \\ u_{I}-R_{I}u_{I-1} \end{array}\right]$$ (4)

We selected a free-form deformation (FFD), widely used in medical imaging, which models motion using a sparse mesh of control points based on hierarchical cubic B-splines [27,28]. The hierarchical approach enables robust multiresolution registration, which provides a sequence of meshes with an increasing number of control points. B-splines have interesting properties, such as positivity, symmetry, compact support and maximal order of approximation [29,30], and are therefore a natural choice for the representation of physiological movements [27].

The nonrigid registration problem searches for the spatial transformation *R*_*i*_:(x,y,z)→(x’,y’,z’), which relates the image domain at the *i*_th_-gate *u*_*i*_ to the image domain at the previous gate *u*_i-1_. This procedure requires interpolation to relate pixel intensity between images, with the result that *R*_*i*_(u_i-1_) comprises both registration and interpolation. The nonrigid registration problem can be expressed as

$$\underset{R_{i}}{\text{min}}\;{\left\|u_{i}-R_{i}\left(u_{i-1}\right)\right\|}_{2}^{2}+\;\rho {\text{C}}_{\text{smooth}}\left(R_{i}\right)$$ (5)

where *C*_smooth_ is a smoothness penalty function, generally in the form of a thin plate of metal bending energy and ρ is a regularization parameter [27,28]. The FFD-based registration method was implemented using the software available in MATLAB Central (Dirk-Jan Kroon; B-spline grid, image and point based registration; 2012, retrieved from http://www.mathworks.com/matlabcentral/fileexchange/20057-b-spline-grid-image-and-point-based-registration), which is based on the Quasi Newton L-BFGS optimization method. It required the selection of several parameters: the regularization parameter ρ that weights the penalty function, the number of grid points, and the number of refinement grids within the hierarchical approach. All results were computed with ρ = 10^−4^ and three refinements, the finest being a uniform grid of 47x47 control points. These parameters were heuristically chosen: doing two and three refinements provided similarly good results while with only one refinement the grid was too coarse and led to larger registration errors; ρ values in the range from 10^−3^ to 10^−4^ led to optimum results while values larger than 10^−2^ led to large registration errors.

#### Split Bregman formulation to solve PRIMOR and PBR problems

We implemented PBR [Eq (2)] and PRIMOR using the Split Bregman method, which efficiently handles L1-based constrained problems [24,31,32]. The Split Bregman formulation separates L2- and L1-norm functionals in such a way that they can be solved analytically in two alternating steps. Constraints are imposed using the Bregman iteration. The part including the L2-norm functionals results in a linear system that can be efficiently solved using iterative methods and the part with the L1-norm functionals is solved using shrinkage formulas. As PBR can be obtained from PRIMOR by making γ = 0, we develop the formulation for the general case of PRIMOR.

To perform the split, we include the new variables *d*_*x*_, *d*_*y*_, *w*, and *t* and formulate a new problem that is equivalent to Eq (3)

$$\begin{array}{l} \underset{v,\;d_{x},\;d_{y},\;w,\;t}{\text{min}}\beta \;{\left\|\left(d_{x},d_{y}\right)\right\|}_{1}+\;\alpha {\left\|w\right\|}_{1}+\gamma {\left\|t\right\|}_{1}\;\text{such}\;\text{that}\;{\left\|F\left(u_{p}+v\right)-f\right\|}_{}^{2}\leq \sigma^{2},\;\\ d_{x}=\nabla_{x}\left(u_{p}+v\right),\;d_{y}=\nabla_{y}\left(u_{p}+v\right),\;w=\Phi v,\;t=T\left(u_{p}+v\right) \end{array}$$ (6)

Eq (6) is easily managed using an equivalent unconstrained optimization approach with constraints imposed by adding a Bregman iteration *b*_*i*_. That is,

$$\begin{array}{l} \underset{v,\;d_{x},\;d_{y},\;w,\;t}{\text{min}}\beta \;{\left\|\left(d_{x},d_{y}\right)\right\|}_{1}+\;\alpha {\left\|w\right\|}_{1}+\gamma {\left\|t\right\|}_{1}+\frac{\mu }{\text{2}}{\left\|F\left(u_{p}+v\right)-f^{k}\right\|}_{2}^{2}+\frac{\lambda }{\text{2}}{\left\|d_{x}-\nabla_{x}\left(u_{p}+v\right)-b_{x}^{k}\right\|}_{2}^{2}+\\ \frac{\lambda }{\text{2}}{\left\|d_{y}-\nabla_{y}\left(u_{p}+v\right)-b_{y}^{k}\right\|}_{2}^{2}+\frac{\lambda }{\text{2}}{\left\|w-\Phi v-b_{w}^{k}\right\|}_{2}^{2}+\frac{\lambda }{\text{2}}{\left\|t-T\left(u_{p}+v\right)-b_{t}^{k}\right\|}_{2}^{2} \end{array}$$ (7)

where μ is a regularization parameter that weights the data fidelity term, λ is a regularization parameter that weights the terms imposing the constraints for the dummy variables, *k* is the iteration number and the Bregman iterations are updated as

bxk+1=bxk+∇x(u+pvk+1)−dxk+1byk+1=byk+∇y(u+pvk+1)−dyk+1bwk+1=bwk+Φvk+1−wk+1btk+1=btk+T(u+pvk+1)−tk+1fk+1=fk+f−F(u+pvk+1) (8)

Since the image variation *v* and the auxiliary variables *w*, *d*_*x*_, *d*_*y*_, and *t* are independent of each other, Eq (7) can now be split into several equations (one for each variable) that are solved sequentially, as follows:

$$\begin{array}{l} v^{k+1}=\underset{v}{\text{min}}\;\frac{\mu }{2}{\left\|F(u_{p}+v)-f^{k}\right\|}_{2}^{2}+\frac{\lambda }{2}{\left\|d_{x}{}^{k}-D_{x}(u_{p}+v)-b_{x}^{k}\right\|}_{2}^{2}+\frac{\lambda }{2}{\left\|d_{y}{}^{k}-D_{y}(u_{p}+v)-b_{y}^{k}\right\|}_{2}^{2}\\ +\frac{\lambda }{2}{\left\|w^{k}-\Phi v-b_{{}_{w}}^{k}\right\|}_{2}^{2}+\frac{\lambda }{2}\;{\left\|t^{k}-T(u_{p}+v)-b_{t}^{k}\right\|}_{2}^{2}\\ d_{x}{}^{k+1},d_{y}{}^{k+1}\underset{d_{x},d_{y}}{=\text{min}}\;\beta {\left\|\left(d_{x},d_{y}\right)\right\|}_{1}+\frac{\lambda }{2}{\left\|d_{x}-D_{x}(u_{p}+v^{k+1})-b_{x}^{k}\right\|}_{2}^{2}+\frac{\lambda }{2}{\left\|d_{y}-D_{y}(u_{p}+v^{k+1})-b_{y}^{k}\right\|}_{2}^{2}\\ w^{k+1}=\underset{w}{\text{min}}\;\alpha {\left\|w\right\|}_{1}\;+\frac{\lambda }{2}{\left\|w-\Phi v^{k+1}-b_{{}_{w}}^{k}\right\|}_{2}^{2}\\ t^{k+1}=\underset{t}{\text{min}}\;\gamma {\left\|t\right\|}_{1}+\frac{\lambda }{2}\;{\left\|t-T(u_{p}+v^{k+1})-b_{t}^{k}\right\|}_{2}^{2} \end{array}$$ (9)

Since the solution of *v* only involves L2-norm functionals, it can be obtained exactly as the solution of the linear system

$$\begin{array}{l} Kv^{k+1}=r_{}^{k}\\ K=\mu F^{T}F+\lambda D_{x}{}^{T}D_{x}+\lambda D_{y}{}^{T}D_{y}+\lambda T^{T}T+\lambda I\\ r_{}^{k}=\lambda D_{x}{}^{T}\left(d_{x}{}^{k}-D_{x}u_{p}-b_{x}^{k}\right)+\lambda D_{y}{}^{T}\left(d_{y}{}^{k}-D_{y}u_{p}-b_{y}^{k}\right)+\lambda T^{T}\left(t^{k}-Tu_{p}-b_{t}^{k}\right)+\lambda \Phi^{T}\left(w^{k}-b_{w}^{k}\right) \end{array}$$ (10)

Note that Eq (10) constitutes a very large-scale problem, where K = N×N and N is the number of pixels, yet it can be solved efficiently using a Krylov solver, such as the biconjugate gradient stabilized method, which involves only matrix-vector multiplications:

$$\mu F^{T}\left(Fv\right)+\lambda D_{x}{}^{T}\left(D_{x}v\right)+\lambda D_{y}{}^{T}\left(D_{y}v\right)+\lambda T^{T}\left(Tv\right)+\gamma v=r$$ (11)

The auxiliary variables *d*_*x*_, *d*_*y*_, *w*, and *t* are solved analytically using shrinkage formulas, which are thresholding operations [24,33].

djk+1=max(sk−βλ,0)|Dj(up+vk+1)+bjk|sk,sk=|Dx(up+vk+1)+bxk|2+|Dy(up+vk+1)+byk|2,j=x,ywk+1=shrink(Φvk+1+bwk,αλ)=max(|Φvk+1+bwk|−α/λ,0)sign(Φvk+1+bwk)tk+1=shrink[T(up+vk+1)+btk,γλ]=       max(|T(up+vk+1)+btk|−γλ,0)sign(T(up+vk+1)+btk) (12)

#### Regularization parameter selection

Regularization parameters related to the Bregman iterations in the reconstruction method (μ and λ in [Eq (7)]) were selected following suggestions from previous studies [30,32]. For μ≤4 the method converged to the same solution for different iteration numbers, which were selected as a number of iterations that yielded minimum mean-square error, considering the ideal high-dose image as the correct solution.

The weighting parameters that control the relative degree of image variation sparsity and spatial and temporal image sparsity were heuristically determined as follows. For PBR [Eq (2)], decreasing α while increasing β (α = 0.2, β = 0.8) results in a patchy-like pattern (due to the high weight of spatial-TV); increasing α while lowering β (α = 0.8, β = 0.2) results in noisy images (due to the low weight of spatial-TV and high weight of the prior). After testing some other intermediate values we decided to choose α = 0.4 and β = 0.2 as a compromise. For PRIMOR we used these same α and β values and verified the effect of γ. Very low γ values (γ = 0.01) led to results similar to those of PBR. Increasing γ imposes gates to be similar, which filters out noise considerably, but also blurs minor image details, as similarity between gates is too enforced. γ values in the range from 0.1 to 1 were a good compromise. We finally selected γ = 0.5 to avoid giving too much weight to temporal sparsity. Table 1 shows a summary of the parameter values used.

**Table 1:** Regularization parameters selected for PBR and PRIMOR methods.

	μ	λ	β	α	γ
**PBR**	2	1	0.2	0.4	0
**PRIMOR**	2	1	0.2	0.4	0.5

### Data Acquisition and Simulation

Algorithms were evaluated using simulations from rodent data acquired in a real scanner. Real data were acquired with the CT subsystem of an ARGUS PET/CT (SEDECAL) scanner, which is a cone-beam micro-CT scanner based on a flat panel detector [34]. In this scanner, the standard protocol for static imaging acquires 8 frames per projection angle of 512×512 pixels (0.2 mm^2^ pixel size) along 360 equispaced angular positions. Respiratory gated studies were carried out by arranging the data into four gates using software-based retrospective gating [5] and reconstructing each of the four gates with an FDK-based algorithm [26]. In order to keep image quality of each gate similar to that of the static image, we acquired another study using a “high dose protocol” with 32 projection images per view, resulting in a fourfold dose increase.

Using the high dose protocol, we acquired rodent data (10-week-old adult female Wistar rats weighing 300 g and anesthetized with isoflurane). To reduce computational time, we selected a smaller field of view (350×350 pixels) and the central slice (extrapolation to 3D would be straightforward). We used this data set as a gold standard. Animals were handled according to the European Communities Council Directive (2010/63/EU) and national regulations (RD 53/2013) with the approval of the Animal Experimentation Ethics Committee of Hospital General Universitario Gregorio Marañón.

In a real acquisition of a respiratory gated study with the ARGUS scanner, the dose (and the image noise) depends on the parameters of the x-ray source (amperage and voltage), the number of angular positions and the number of frames per angle. To generalize the effect of these parameters on the resulting image to any scanner, we define two figures: dose, given by the number of photons emitted by the x-ray source, and number of projections.

We simulated seven different scenarios: low-dose scenarios, with 120 projections and reducing the number of photons emitted by the x-ray source (I_0_ = 4.5×10^4^, I_0_/2, I_0_/4 and I_0_/6), and subsampled scenarios, by reducing the number of projections per gate (120, 80, 60 and 40 projections) for number of photons I_0_. I_0_ was chosen so as to obtain a noise figure for the prior image similar to that of a real high-dose gate.

Noise was added by modelling the measurements f_i_ as independently distributed Poisson random variables:

$$f_{i}\sim Poisson\left\{\bar{f_{i}}\right\}\begin{array}{cc} & i=1,\ldots ,M \end{array}\text{ with }\bar{f_{i}}=I_{o}e^{-\int u(x,y,z)}$$ (13)

where *u*(*x*,*y*,*z*) is the high-dose reconstruction, *I*_0_ is the number of photons emitted by the x-ray source, and M is the number of measured projections. Then, we simulated respiratory gating by randomly choosing a small number of projections from each gate, and classifying each projection as to pertaining to a specific respiratory phase.

In order to generate statistical results, we generated five different noise realizations for each low dose scenario and five random selections of projection angles for each of the subsampled scenarios. Table 2 shows the 40 simulated data sets, where each scenario is defined by the dose (compared to the dose used in the protocol for static imaging) and the number of projections per gate.

**Table 2:** Simulated data sets used for the evaluations.

Scenario	Dose	# projections per gate	# noise realizations	# random selection of projections cases
High dose protocol (gold standard)	4I_0_	360	1	1
Static protocol 1	I_0_	120	5	1
Static protocol 2	I_0_	120	1	5
Low-dose	I_0_/2	120	5	1
Low-dose	I_0_/4	120	5	1
Low-dose	I_0_/6	120	5	1
Subsampled	I_0_	80	1	5
Subsampled	I_0_	60	1	5
Subsampled	I_0_	40	1	5

Simulations were computed using the IRT code (J A Fessler, Image reconstruction toolbox [IRT], 2011, retrieved from <http://www.eecs.umich.edu/~fessler/code/index.html>).

**Fig 3:**
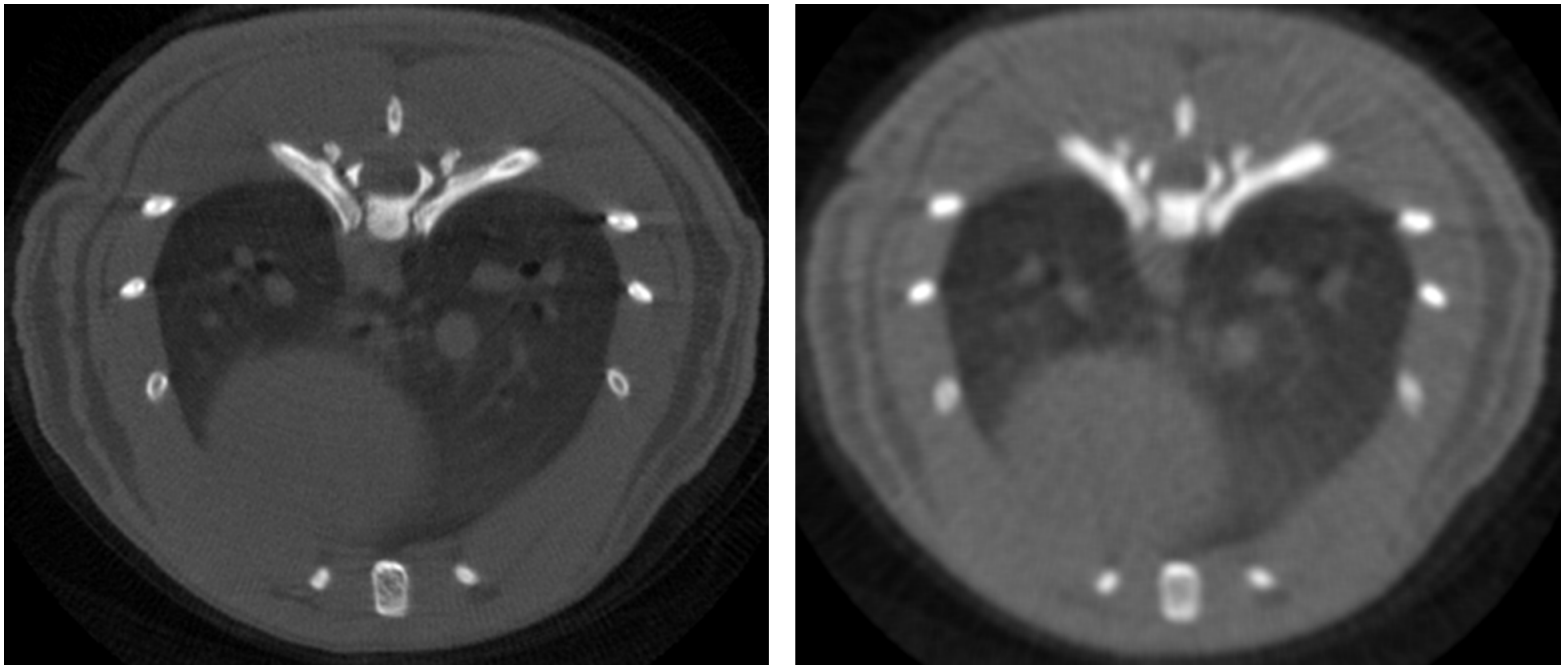
Axial view of respiratory gated data. Left: FDK reconstruction of gated ideal high-dose data for frame 1. Right: Prior image obtained with a filtered version of the FDK reconstruction of the average of the whole data set for a subsampled scenario (120 projections and number of photons I_0_ = 4.5×10^5^).

The prior image was obtained by adding all gates and applying a Gaussian filter with a window of 5 pixels and standard deviation of 3 (right panel of Fig 3).

### Comparison of Methods and Analysis of Images

#### Analysis of the influence of dose and number of projections

**Fig 4:**
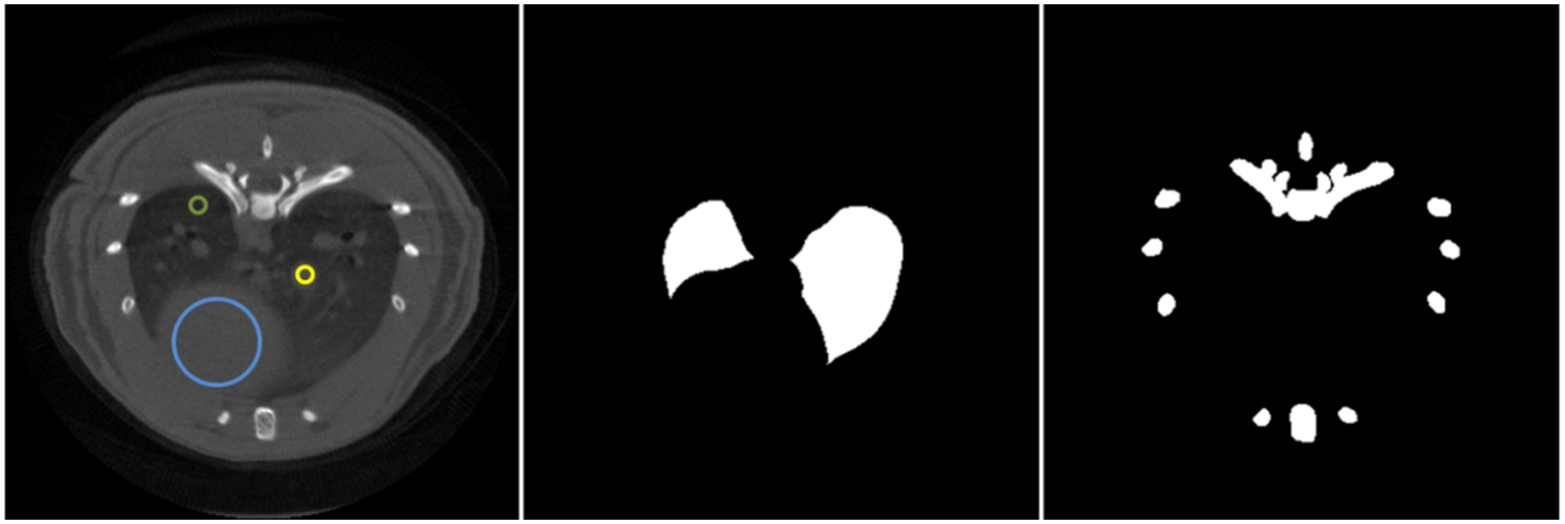
Masks used to measure contrast-to-noise ratio and MSE. Left: Masks used to measure contrast-to-noise ratio as the absolute difference between the yellow and green ROIs divided by the noise measured in the blue ROI. Middle: Masks to compute MSE in the lung area. Right: Masks to compute MSE in the bone area.

Data were reconstructed with PBR and PRIMOR. To evaluate the influence of dose and number of projections for each method, images were compared in terms of four metrics: 1) mean-square error (MSE) in bone; 2) contrast-to-noise ratio in lung (ROIs shown in Fig 4); 3) total variation of the difference between reconstructed images and the target; and 4) solution error norm (SEN). Five different realizations were created for each scenario, thus yielding a total of 40 data sets. To evaluate the statistical significance of the difference between PBR and PRIMOR we used a Mann-Whitney test, as it is more robust and avoids the assumption of normality in the data.

We assessed the recovery of bone tissue by computing the MSE of a ROI in the bone area with respect to the same ROI in the reference high-dose image. Bone region was delimited by using a mask that comprises bone and some of the surrounding tissue in order to account for blurring artefacts. Noise was measured as the standard deviation in a circular ROI drawn in a homogeneous region within the heart. Contrast-to-noise ratio was assessed as the absolute difference between the HU in a lung nodule and the value in healthy lung tissue divided by the noise in the heart. All masks are shown in Fig 4.

Artifacts were assessed by the streak artefact indicator (SAI), which measures the total variation of the difference between reconstructed images and the target. It has been previously used to measure artefacts in CT [35]. Convergence was assessed using the solution error norm, computed in the entire image and using high-dose image as a reference.

Finally, image texture was evaluated by visual inspection. We provide videos of the reconstructed images for different scenarios, which help to appreciate the motion between different respiratory gates (videos can be found at https://github.com/HGGM-LIM/prior-motion-reconstruction-CT).

#### Correction of respiratory motion

Compensation for respiratory motion was assessed by drawing a profile across an area of soft tissue that presented large movement across respiratory gates and comparing with the same profile for high-dose FDK images.

#### Application study

A possible clinical application would be to assess the degree of infection in lung diseases such as tuberculosis, which is generally based on the inspection of images to quantify the density and number of nodules in the lung.

Since the nodules in tuberculosis have a similar contrast and shape than vessels in an axial slice, we considered vessels as a good surrogate of granulomas and performed the quantification on them. To this end, we used a previously available semiautomatic tool based on region growing for granuloma quantification in tuberculosis. With this tool, an expert segmented five small vessels in all the studies: high dose image (used as a reference) and FDK, PBR and PRIMOR images, for the different scenarios. We then computed the Jaccard similarity index of the masks obtained with the three methods, as compared to those obtained with the reference.

## Results

### Analysis of Influence of Dose and Number of Projections

**Fig 5:**
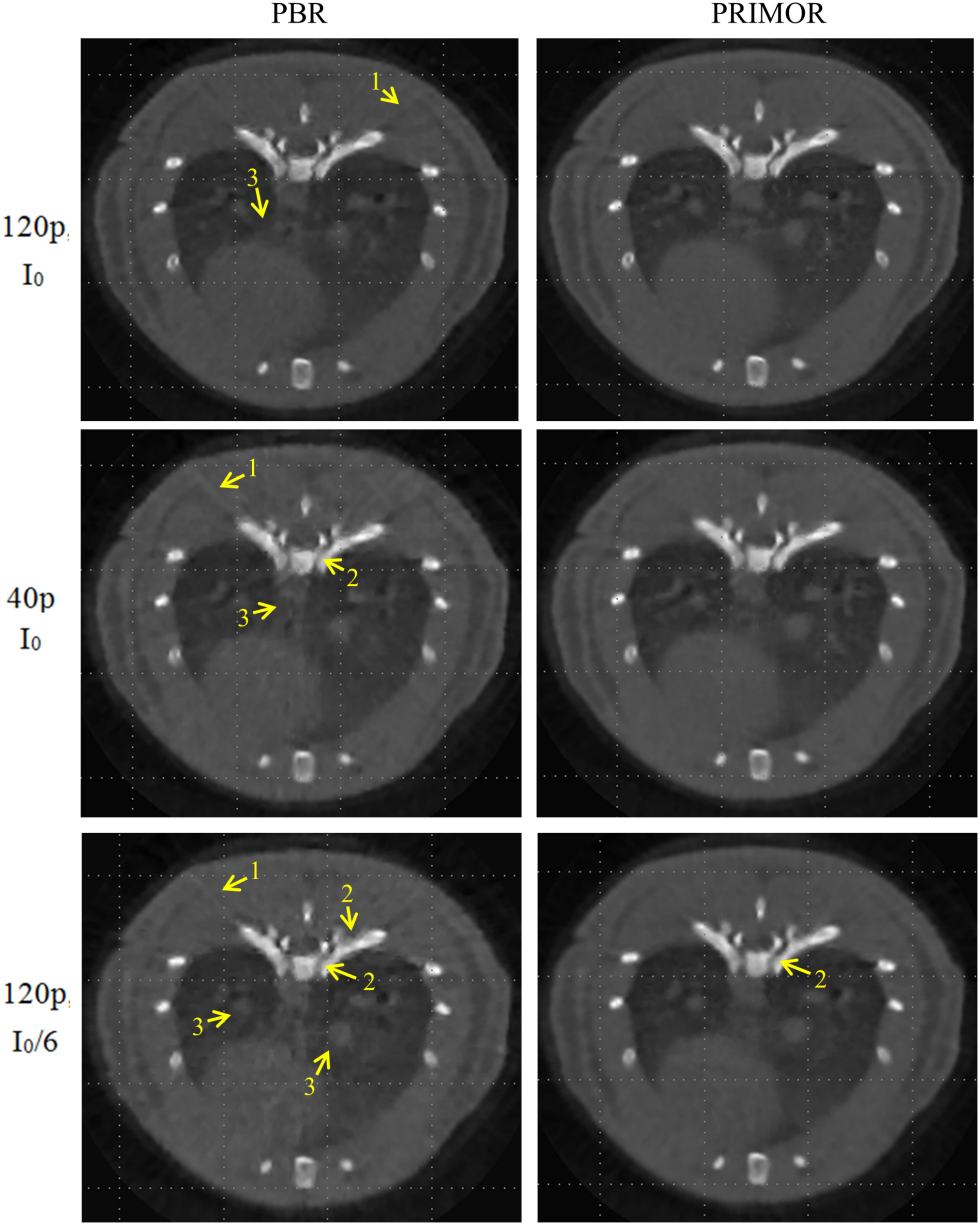
Images corresponding to different scenarios for respiratory phase three, reconstructed with PBR and PRIMOR algorithms. From top to bottom, each row represents a different scenario: 120 projections and dose corresponding to a maximum number of photons I_0_ (subsampled scenario), 40 projections and I_0_ (subsampled scenario), and 120 projections and number of photons I_0_/6 (low-dose scenario). Yellow arrows indicate where artifacts are more noticeable: an increase in streak artifacts (1), blurred edges for bone tissue (2), and blurred edges for soft tissue (3). Videos for these results showing images for all gates are available at https://github.com/HGGM-LIM/prior-motion-reconstruction-CT.

Fig 5 shows the results of PBR and PRIMOR for different noise levels and number of projections for respiratory phase three. For the best case scenario (120 projections and maximum number of photons I_0_) (first row in Fig 5), PBR leads to slightly blurred edges for bone and soft tissue and to a decrease in contrast within bone tissue, while PRIMOR is almost unaffected. When the number of projections decreases (second row in Fig 5), PBR leads to streak artefacts and to highly blurred edges for bone and soft tissue, while PRIMOR removes most of these artifacts. When the dose decreases (third row in Fig 5), PBR leads to noisier images, which affects both bone and soft tissue, and distort nodules in the lung, while PRIMOR is just affected by a slight blur in bone tissue. Overall, PRIMOR leads to improved images for all scenarios. Videos showing reconstructed images of the four respiratory gates for the different scenarios are available at https://github.com/HGGM-LIM/prior-motion-reconstruction-CT.

**Fig 6:**
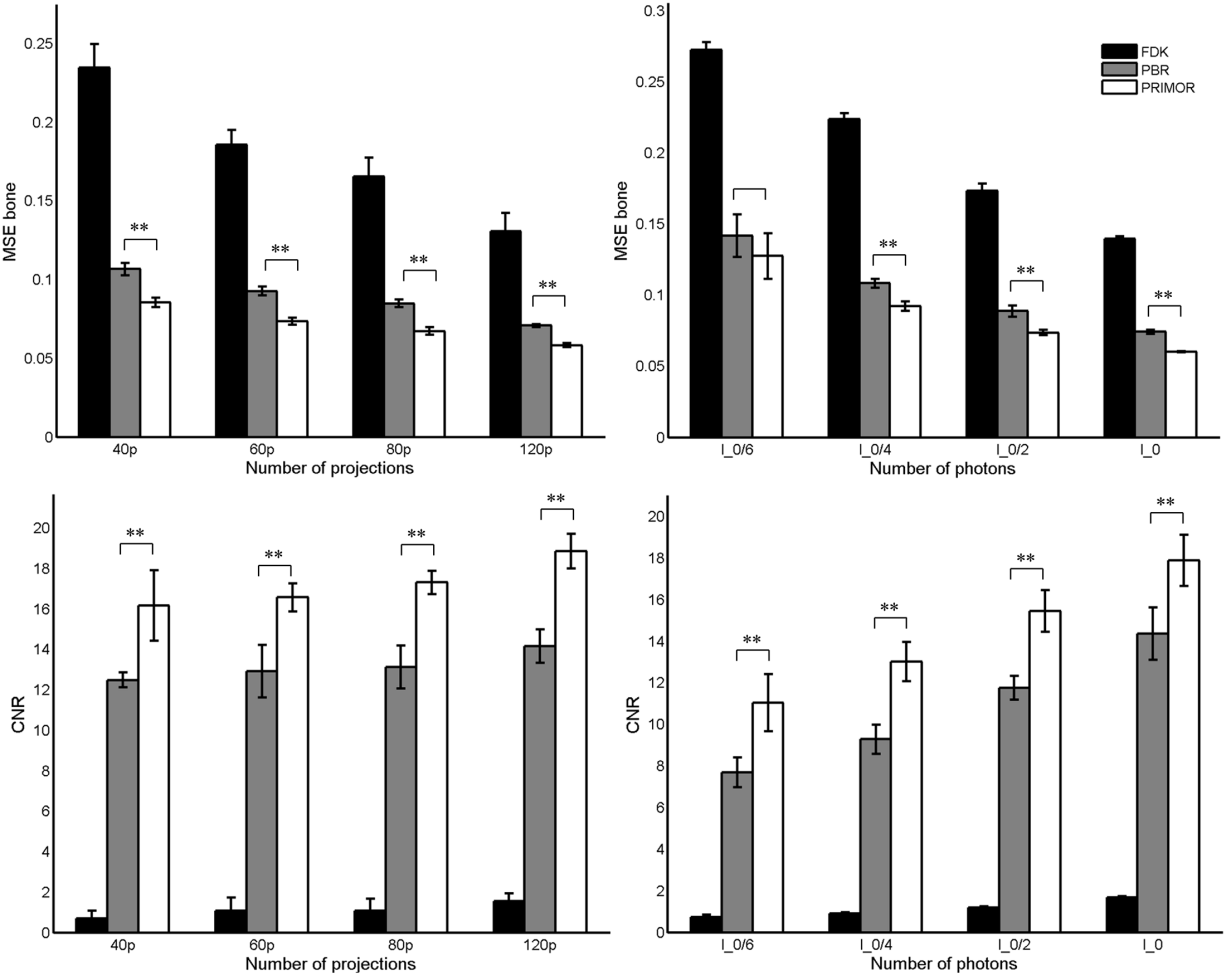
Mean-square error in the bone area (top) and contrast-to-noise ratio inside the lungs (bottom) in the images reconstructed with FDK, PBR and PRIMOR for the different scenarios. Mean and SD of five noise realizations are provided for each scenario. The left panels show different numbers of projections (40, 60, 80 and 120 projections) for a dose corresponding to a number of photons I_0_ = 4.5×10^4^; the right panel represents the different dose values (I_0_, I_0_/2, I_0_/4 and I_0_/6) for 120 projections.

Fig 6 shows the MSE in the bone area and the contrast-to-noise ratio in the lung area for all the different scenarios. Both iterative methods greatly improve FDK reconstruction in all cases. For all subsampled scenarios PRIMOR provided significantly better contrast recovery and less MSE than PBR (p<0.01). Compared with PBR, PRIMOR obtained similar values of MSE for half number of projections and better CNR for one third of the projections. When lowering the dose, PRIMOR also produced significantly better contrast recovery and less MSE than PBR (p<0.01) for all cases except for the case of lowest dose (I_0_/6), in which PRIMOR presents lower MSE but is not statistically significant. Overall, PRIMOR yielded similar values of MSE and CNR than PBR for a two to threefold reduction in dose or number of projections.

**Fig 7:**
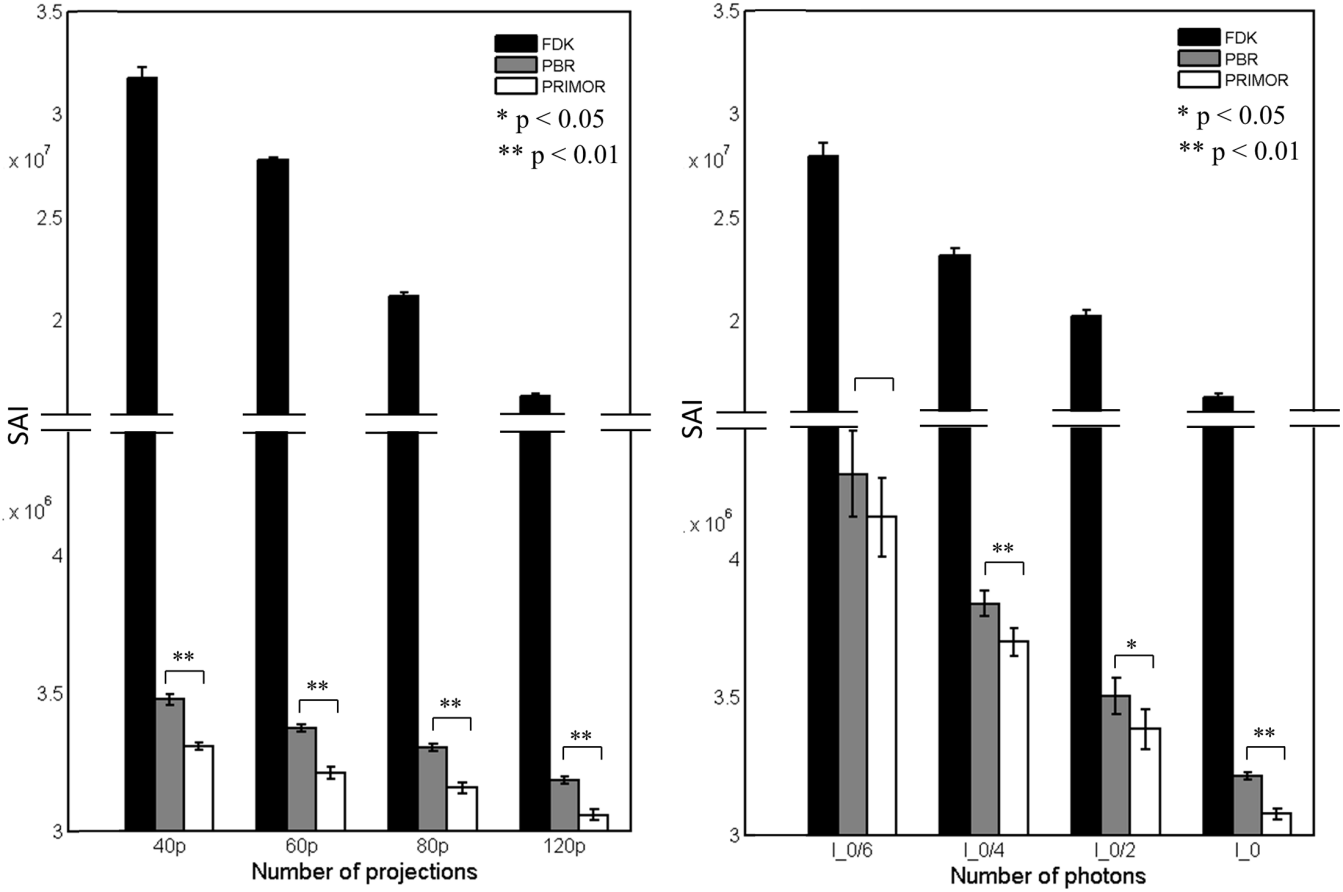
SAI computed as total variation of the difference between reconstructed images and the target for different number of projections (left) and number of photons (right). Graphs show mean values and standard errors.

Fig 7 shows the SAI, computed as total variation of the difference between reconstructed images and the target. For different number of projections, PRIMOR presented significantly lower SAI than PBR (p<0.01). For different dose values, PRIMOR also presented lower SAI than PBR, statistically significant in all cases (p<0.05) except for the case of lowest dose (I_0_/6). The effect of dose was less noticeable than that of number of projections. In all cases both PBR and PRIMOR led to much lower SAI than FDK.

**Fig 8:**
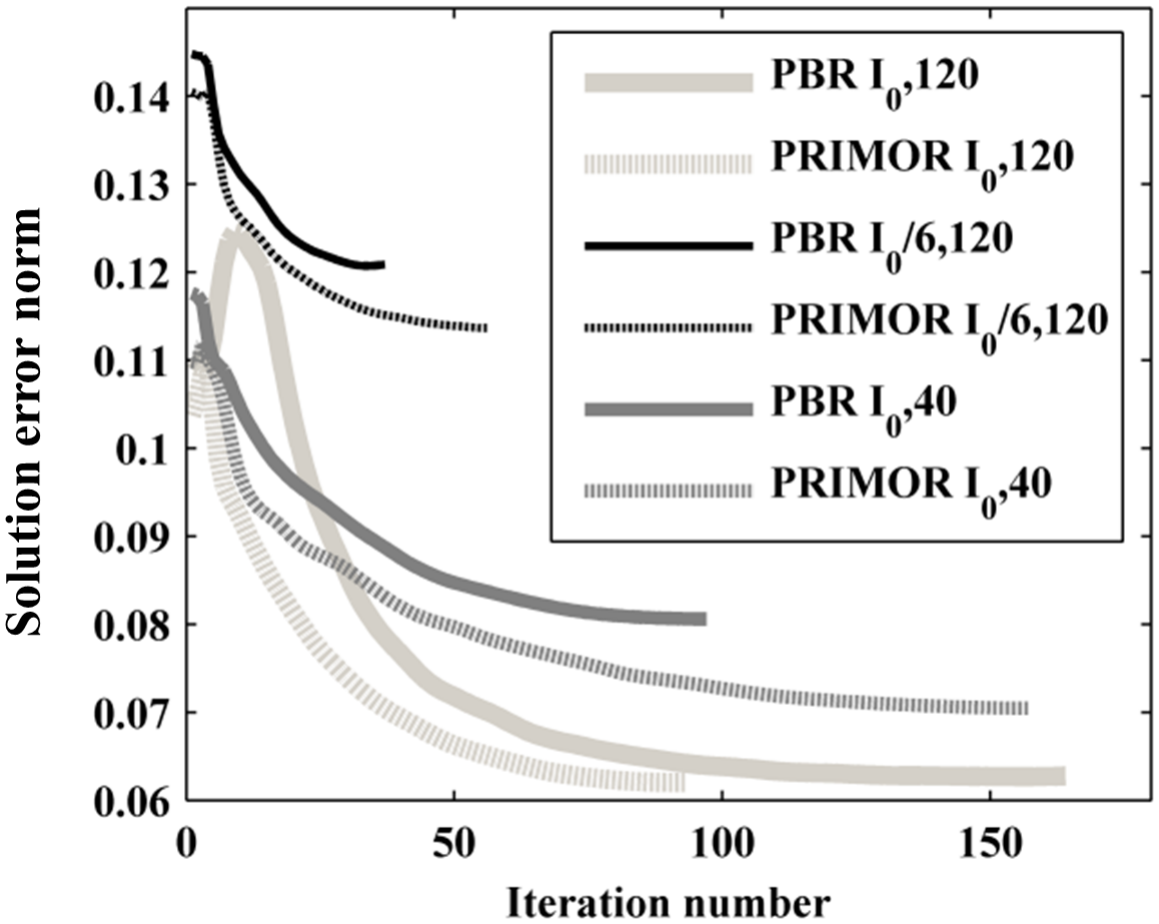
Convergence of PBR and PRIMOR algorithms for different scenarios. Relative MSE vs. the iteration number given by both PBR and PRIMOR methods for the static imaging protocol (I_0_ = 4.5×10^4^, 120 projections per phase), a decrease in dose (I_0_/6, 120 projections) and decrease in the number of projections (I_0_, 40 projections).

Fig 8 shows relative MSE vs. iteration number obtained by PBR and PRIMOR methods for the different scenarios. PRIMOR presented lower error in all cases, where differences were most significant for low dose. Regarding convergence, PRIMOR generally converged at higher iteration number but with lower error than PBR.

### Analysis of Compensation for Respiratory Motion

**Fig 9:**
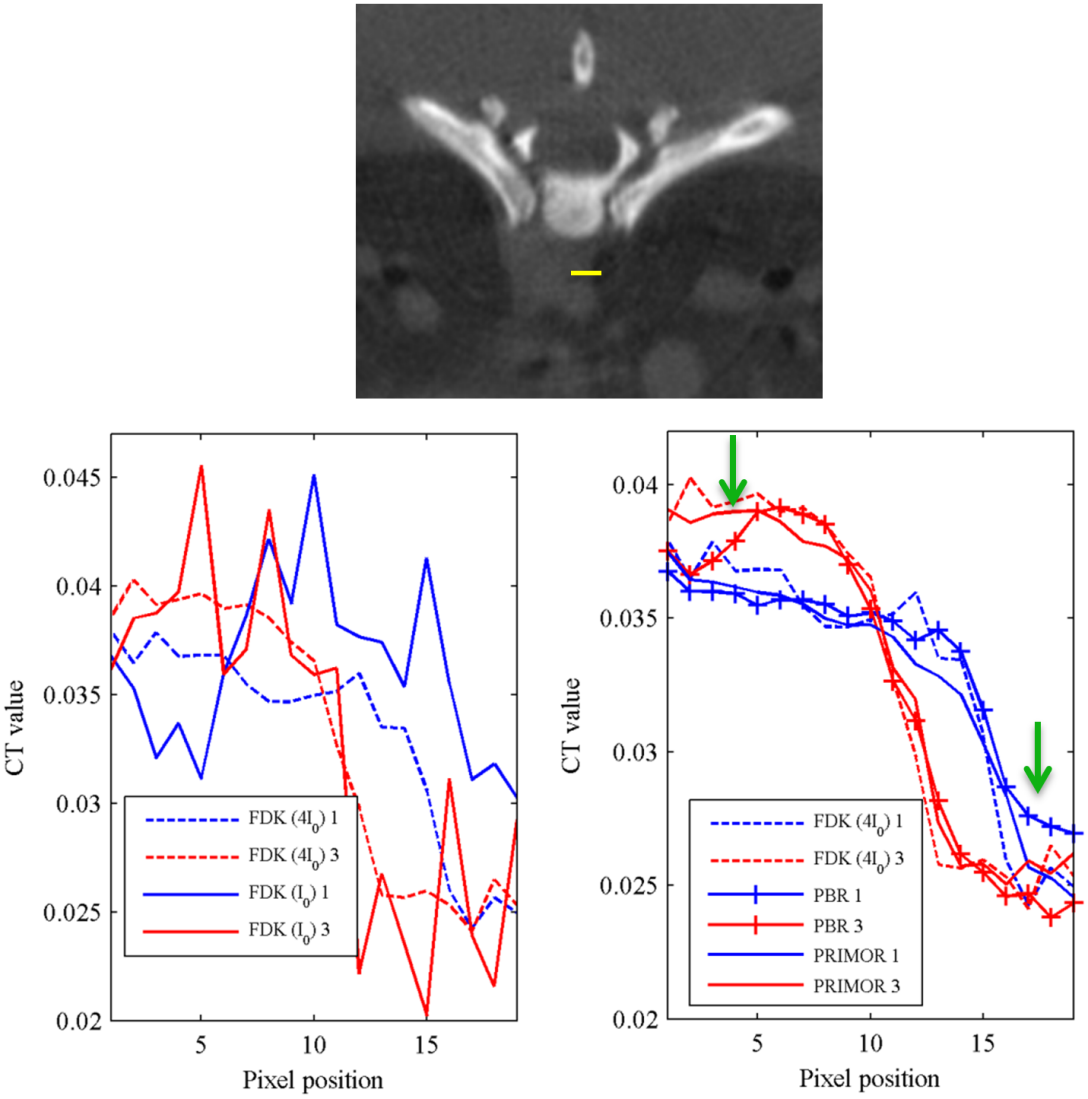
Respiratory motion correction. Normalized profile along the yellow line in the top figure for ideal high-dose FDK (4I_0_ and 360 projections) and for a subsampled scenario (120 projections and I_0_) reconstructed with FDK (left) and with PBR and PRIMOR corresponding to respiratory gates 1 and 3. Green arrows show where differences between PBR and PRIMOR are more noticable.

Fig 9 shows a profile along a line containing soft tissue that presented large motion for the ideal high-dose FDK and for FDK, PBR and PRIMOR reconstructions of respiratory gates 1 and 3 using 120 projections and a number of photons I_0_ = 4.5×10^4^. The profiles reveal the presence of respiratory movement for the two respiratory gates. Although both PBR and PRIMOR can improve FDK reconstruction by correcting the movement artifact between the different gates, profiles on PRIMOR reconstructions are more similar to those of the high dose FDK. This is confirmed by the videos mentioned in Fig 5, which show how PBR is able to correct the motion artefact, recovering the difference between gates, but it is more severely affected by noise and other artefacts than PRIMOR.

### Application Study

**Fig 10:**
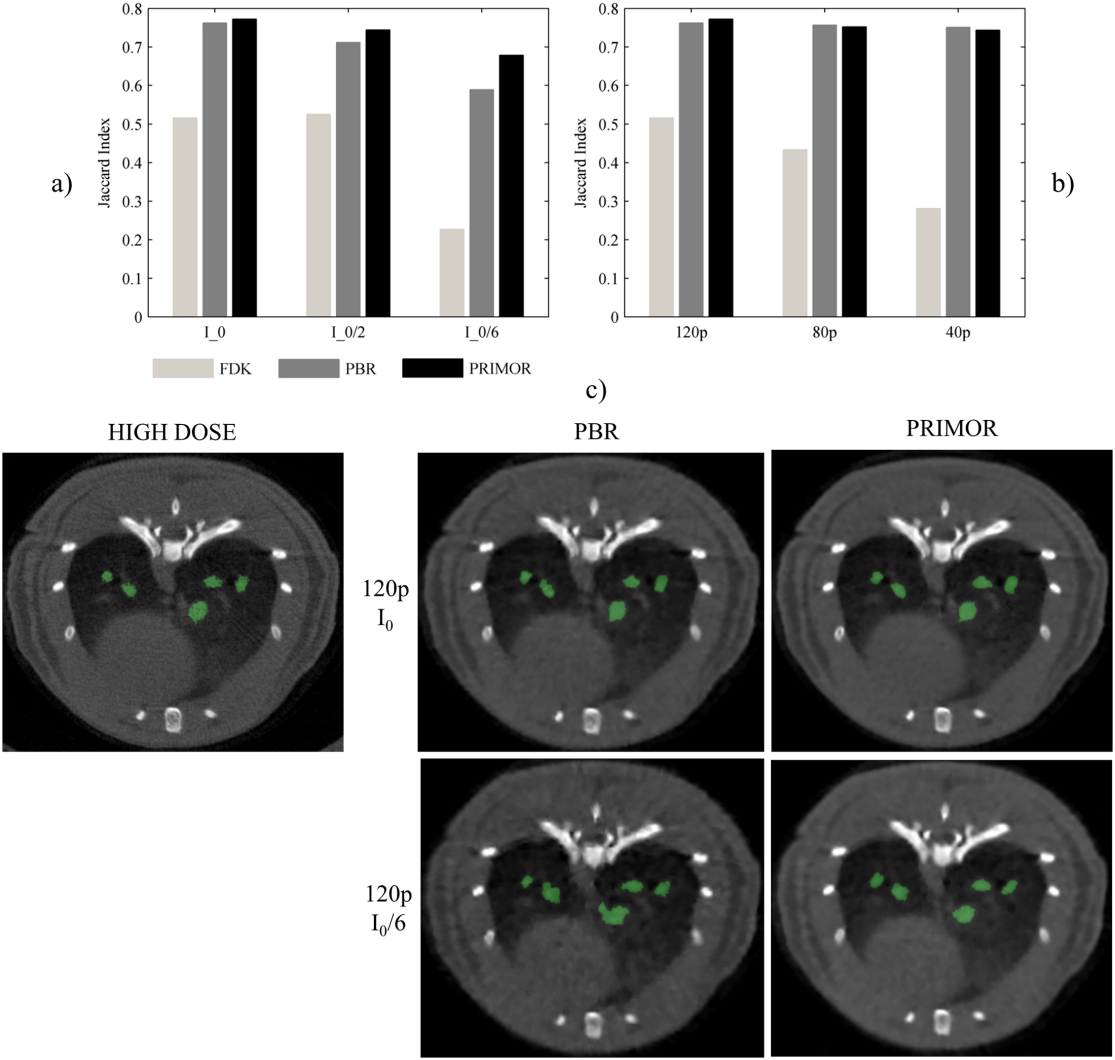
Assessment of the influence of the different scenarios and reconstruction methods on the segmentation of lung tissue. Jaccard index for different dose (a) and number of projections (b) when using FDK, PBR and PRIMOR methods. C) Example of the segmented masks from high dose image and PBR and PRIMOR methods for the best (120 projections and I_0_) and worst case scenarios (120 projections and I_0_/6).

Lowering the dose led to a substantial decrease in Jaccard similarity index for FDK, while PBR and PRIMOR were less affected. PRIMOR led to higher similarity index than PBR in all cases (Fig 10a and 10b). Decreasing the number of projections showed a similar effect when using FDK, while it did not affect PBR and PRIMOR. Fig 10c shows an example of the segmented mask on the high dose image (used as the reference) and masks obtained from PBR and PRIMOR reconstructions for the best case scenario (120 projections and I_0_) and worst case scenario (120 projections and I_0_/6). At lower dose, the segmentation from PBR is clearly worse than the segmentation from PRIMOR, due to the presence of artefacts.

### Computation Time

The code was implemented in MATLAB and run on a Linux machine with 16 CPU of 64-bit and 2.3 GHz and with 16 GB RAM. We used a straightforward parallelization of PRIMOR over the four gates to reduce computation times. Motion estimation took 1.9 min. The reconstruction step took 90 s per iteration, out of which 81 s were employed for solving the linear system and 7 s for the Bregman iteration. As the linear system does not need to be solved with high precision, a tolerance of 10^−2^ was sufficient and provided the same solution as 10^−4^, which took 145 s. The total computation time was 200 min for 98 iterations for the best case scenario (120 projections and I_0_) and 81 min for 55 iterations for the worst case scenario (120 projections and I_0_/6).

## Discussion

Commercial scanners use traditional FDK for image reconstruction, but it results in severe artifacts when reconstructing respiratory-gated CT data using standard dose. To ensure a sufficient number of projections per gate, a dose four times higher may be needed (for four gates). An alternative is to use a PBR method which has been shown to reduce artefacts when there are insufficient numbers of projections. We propose PRIMOR, a novel PBR and motion-based compressed sensing method for reducing the radiation dose in CT respiratory gating.

We compared PRIMOR with FDK and PBR, an analogous prior-based method with no estimation of motion, in scenarios corresponding to different dose levels and numbers of projections. PRIMOR produced better results in all scenarios by reducing streak artifacts, reducing noise, and improving image quality in terms of MSE and contrast-to-noise ratio. Adopting PBR as a reference, PRIMOR increased CNR in up to 33% and decreased MSE, streak artifact indicator and solution error norm in up to 20%, 4% and 13%, respectively. PRIMOR also presented better compensation for respiratory motion and more accurate segmentation of lung tissue.

Few methodologies have previously addressed the lack of projections in respiratory and cardiac gated micro CT. Some methods are based on filling the missing data by using a prior image built from the data. This is the case of the least-error sorting technique [35], which ensures that all angular positions are filled, and the method presented in [36], which fills the missing regions in the Fourier domain. McKinnon-Bates method also used the average of all respiratory gates to construct the prior image [37,38]. However, methods based on compressed sensing show improved results with respect to these previous methods. In a chest phantom, PICCS outperformed both FDK with McKinnon-Bates correction and spatial TV [39]. In a previous study we showed that the use of the wavelet transform in the prior term instead of the gradient commonly used in PICCS led to a more natural texture in the image [16].

The exploitation of temporal sparsity based on spatiotemporal total variation reconstruction yielded better results than McKinnon-Bates and low dose phase-correlated reconstruction on a mouse phantom [40]. A variation of the previous method, weighted spatiotemporal TV, improved results based on a spatial adaptive weighting function that assigns larger weight to regions without motion. This technique enabled the reconstruction of retrospective cone beam micro-CT data using only between 55 and 95 projections per angle [41]. Comparing our method to PICCS and spatiotemporal TV, PRIMOR is an extension of these two methods, as it combines PICCS with motion based reconstruction methods, which is an improvement with respect to spatiotemporal TV as it accounts for motion between consecutive gates. PICCS assumes that the difference between the different gates and the prior image (the mean of all gates) is sparse. PRIMOR provides a sparser transform by modelling the differences between gates. As PRIMOR combines both PBR and motion-based methods, it achieves the benefits of both methods. On the one hand, the prior image helps to maintain the image texture while on the other hand a sparser transform restricts the solution space, which results in a reduction of artefacts. Regarding the weighted spatiotemporal TV method [41], it is possible to incorporate this strategy into the PRIMOR method by including a similar spatial adaptive weight function, perhaps further improving results.

As for motion estimation, we used a free-form deformation method based on hierarchical B-splines [27]. B-splines provide a smooth natural choice for representation of physiological movements, and the hierarchical approach enables robust registration. These properties have made this registration approach to be widely used in medical imaging. We found the algorithm very easy to use by controlling the smoothness with the number of control points and the registration error with a thresholding parameter. Previous motion-based reconstruction methods in magnetic resonance imaging have found phase-based motion estimation to be better than previously used block-matching and optical flow methods [17]. Other possible nonrigid registration algorithms are Kanade algorithm or Demons registration algorithm [42,43], which are based on optical flow methods. While we have shown that PRIMOR gains by including a model of motion within the reconstruction algorithm, we will not expect further improvements by using other nonrigid registration algorithms. However, this is limited to our data sets and further work would be required to better understand the differences between motion estimation methods for low-dose CT.

Few previous studies have combined prior-based reconstruction methods with registration in CT [20,23]. These works aimed to improve image quality in low dose acquisition using a previously acquired high quality prior image which is not registered with the data, so a registration step was included by modifying the prior penalty term. There are two substantial differences with our work: first, we are considering the case in which we do not have a high quality previous acquisition image; second, the built prior is a blurred image made from averaging all un-gated data. Thus, in our work, instead of modifying the prior penalty term, we include in the cost function a new penalty term that takes into account motion between consecutive gates, considering that there would be little difference among them.

The present study is subject to several limitations. Although we evaluated the methods at different dose levels and with different numbers of projections, further validation with experimentally acquired data is required. Nevertheless, major differences are not expected. Another limitation is that results shown were obtained for a set of user-determined parameters values. Parameter selection was required for the registration algorithm, the Split Bregman method, and the Gaussian smoothing of the prior image. We confirmed that similar results were obtained when varying these parameters within a range. The regularization parameter weighting the penalty function in the registration step was found to yield similar results for a wide range of values. The parameters μ and λ weighting the Bregman iteration terms were chosen following the suggestions from previous studies [24,32]. Goldstein and Osher [24] proved that the final outcome is independent of these parameters as long as they are sufficiently small. Low values ensure that noise is removed in the first iterations, finer scales of the image are recovered sequentially from coarser to finer as the iteration number increases, and noise is recovered the last. Abascal *et al*. [32] showed that, in practice, one can initially select small values for these parameters to ensure convergence and then increase μ (weighting of the data fidelity term) to achieve faster convergence. The most important parameter is the number of iterations, as one must stop before fitting the noise. In this work we have selected a number of iterations that led to the minimum solution error, in order to compare the best possible solution obtained by both PBR and PRIMOR methods. In practice, it may be convenient to select a small μ value that leads to slow convergence, and the solution will be similar for a wide range of number of iterations, as previously reported [19]. The parameters α and β, which control the relative degree of sparsity, were also heuristically chosen to provide best results for PBR. We used these values for PRIMOR and then verified the effect of γ. Higher γ values (higher weight to temporal sparsity) improved image texture. Conversely, very large values may lead to miss minor image details from specific gates, as similarity between gates is enforced. Our trade-off was to choose a relatively small γ, to make sure results were not biased. As future work, finding an optimum γ value may further enhance results with respect to PBR.

Besides, further improvements could be made to the proposed method. For instance, Ramani and Fessler [44] included statistical data modeling, which improved convergence. Furthermore, we used a prior image based on the average of data for all phase bins, although other priors, such as a running average, could yield better results [13].

Regarding the execution time, the most computationally expensive parts of the algorithm are the projection and backprojection operators. In this work, these operations were computed using the IRT code (J A Fessler, Image reconstruction toolbox [IRT], 2011, retrieved from <http://www.eecs.umich.edu/~fessler/code/index.html>). The projection of one slice (350×350) using IRT code took 1.8 s. We are working on a GPU implementation where the projection of a volume (350^3^) takes 0.95 s, thus supporting the idea that with this implementation the algorithm is suitable for practical applications.

In conclusion, we propose PRIMOR, a novel method for the reconstruction of low-dose CT data with respiratory gating, which improves previous PBR methods by including a new penalty based on the model of motion between consecutive respiratory gates. The proposed method shows an improvement in image quality and allows a reduction of dose or number of projections of two to three times with respect to previous PBR approaches.
